# Does Saffron Have Antisolar and Moisturizing Effects?

**Published:** 2010

**Authors:** Shiva Golmohammadzadeh, Mahmoud Reza Jaafari, Hossein Hosseinzadeh

**Affiliations:** a *Nanotechnology Research Center,*; b * Biotechnology Research Center,*; c * Pharmaceutical Research Center and School of Pharmacy, Mashad University of Medical Sciences.*

**Keywords:** Saffron, Antisoloar, Sun protection factors, Moisturizing

## Abstract

The objective of this study was to investigate the effects of saffron as a natural sunscreen and moisturizer. The pollens of the saffron were dried and powdered in a grinder. The experimental formulations included a homosalate (8%) lotion reference, lotions with 2, 4 and 8% of grinded saffron, and the control lotion base without saffron. The lotions containing saffron were prepared like homosalate lotion reference according to FDA. The sun protection factors (SPFs) of the formulations were determined by an *in vitro *spectrophotometry method. The moisture contents of the skin before application and after 30 min and 3, 5 and 7 h post-application of the formulations were measured in human volunteers using Corneometer. The results indicated that, there was no significant difference between the SPF values of the 4% saffron lotion and the homosalate lotion reference. However, the SPF of 8% saffron lotion was significantly more than that of homosalate lotion reference. These results showed that in equal concentrations saffron lotion could act as a better antisolar agent compared to homosalate. Furthermore, there were no significant differences in skin moisture saffron lotions and the control lotion without saffron during the 7 h post-application period.

Saffron can be used as a natural UV absorbing agent. The 4% saffron lotion showed an SPF value equivalent to the 8% homosalate lotion reference by an *in vitro *method. There were no significant differences of skin moisture contents after application of the saffron lotions and the control base lotion without saffron**. **

## Introduction

Every year, more than one million people are diagnosed with skin cancer and about 10,000 die from malignant melanoma. Most skin cancers occur on the areas that are most frequently exposed to the sun, such as the face, neck, and the back of the hands (1). The harmful effects of solar radiation are caused by the ultraviolet (UV) part of the solar rays, which can be divided into three regions: UVA, from 320 to 400 nm; UVB, from 290 to 320 nm and UVC, from 200 to 290 nm. UVA has been further subdivided into short-wave (UVAII, 320–340nm) and long-wave (UVAI, 340–400nm). Among these radiations, UV-A and UV-B are mainly responsible for skin pathologies such as sunburns, cutaneous degeneration, photosensitivity, phototoxicity, actinicelastosis, photo-aging, immunosuppression and skin cancer (2- 4). UVC radiation is filtered by the atmosphere before reaching the earth (4).

It has been known for decades that sunscreens are capable of protecting man from harmful effects of solar radiation (3, 5). Sunscreen products contain UV filters, which absorb the UV radiation (chemical UV filters), or reflect and scatter the UV radiation (physical UV filters). The inorganic compounds titanium oxide and zinc oxide are commonly used as physical UV filters. However, a wider range of organic compounds are used as chemical UV filters (6). Due to these facts, the sunscreens substances are now incorporated into everyday products such as moisturizers, creams, lotions, shampoos, mousses, and other hair and skin preparations. The regular use of these products may help to reduce the chance of the harmful effects of ultraviolet radiation (4). 

The efficacy of a sunscreen is usually expressed by the sun protection factor (SPF). Higher SPF values result in more effective products in preventing sunburn. Recent studies have provided evidence that sunscreens are absorbed systemically following topical application to the skin (7, 8). Sunscreen products are usually applied superficially to large skin areas; therefore, penetration of the sunscreen occurs, which is not desirable (7). Nowadays, because of the benefits of products containing natural compounds, acceptable of these products by the users, also the probability of the systemic absorption, using natural products that can absorb ultraviolet radiation is of great interest (9).

Natural substances extracted from plants have recently been considered as potential sunscreen resources because of their ultraviolet absorption in the UV region and their antioxidant activity (9-14). 

Saffron is the dried stigmas of a flower scientifically identified as *Crocus sativus *L. Saffron is a perennial stemless herb widely cultivated in Iran and some other countries such as India, Spain and Greece. Commercial saffron comprises the dried red stigma with a small portion of the yellowish type attached (15). Pharmacological studies have been revealed that saffron extracts or its constituents have antitumor (16- 18) and hypolipidemic effects as well as radical scavenging properties (19). Antinociceptive, antiinflammatory (20), anticonvulsant (21), and antidepressant effects have also been reported in animals (22, 23) and human (24). In traditional medicine, the stigmas of this plant have been used as antitussive and expectorant (15).

There is strong evidence that the DNA-damaging ultraviolet radiation induces the accumulation of UV-absorbing flavonoids and other phenolics in dermal tissues of the plant. This suggests a physiological function, yet speculative in light protection in plants and of course in human (14, 25).

In this study, because of the advantages of saffron besides having many aromatic and flavonoid compounds such as kaempherol and quercetin, the possibility of using it as a UV radiation absorbent in sunscreen products was evaluated (9, 26). This plant was chosen to be evaluated with regard to the UV absorption properties to be incorporated into the suncare products. 

Another effect of a sunscreen product should be producing a moisturizing effect. The corneometers have gained worldwide acceptance as an efficient instrument to measure the water content in stratum corneum. It can show the dryness of the skin and the effects of treatment (27, 28). 

The aim of this research was to determine the SPF values of the sunscreen emulsions containing saffron by an in vitro method and evaluate the moisturizing effects of saffron formulations on the skin of human volunteers using Corneometer. 

## Eexperimental

*Reagents and samples*

Homosalat and ethanol (analytical grade) were purchased from Merck (Germany). Lanolin, white petrolatum, stearic acid, propylparaben, methylparaben, disodium EDTA, propylene glycol and triethanolamine were purchased from Sigma (USA)*. Crocus sativus *L. stigmata were collected from Ghaen (Khorasan province, Northeast of Iran). 

*Apparatus*

A Shimadzu UV-1700 UV/Visible spectrophotometer, equipped with 1 cm quartz cell, computer and Epson LQ-300 printer were used. Corneometer CM 825, which was mounted on a Multi Probe Adapter® MPA5 (Courage and Khazaka, Electronic GmbH, Cologne, Germany) was used for the evaluation of the skin moisture content.

*Preparation of the reference homosalate lotion as the standard sunscreen*

A standard sunscreen formulation was needed for ensuring reproducible results in SPF determinations. This standard was prepared according to the FDA and Australian standards. According to FDA, the SPF of this standard preparation is 4.47 ± 1.279 (21, 27). Homosalate (8%), lanolin (5%), white petrolatum (2.5%), stearic acid (4%) and propylparaben (0.05%) were heated at 77 - 82°C as the oil phase. Methylparaben (0.1%), disodium EDTA (0.05%), propylene glycol (5%), triehanolamine (1%) and water up to 100% were heated as the aqueous phase with constant stirring until complete solution of each part. The aqueous phase was added to oil phase and the mixture stirred until it cooled down to room temperature (29, 30, 31). 

*Preparation of oil/water emulsion of saffron*

The pollen of *Crocus sativus *was collected from Ghaen in Khorasan province in eastern part of Iran. This province is the main producer of saffron in the world. The pollens of the saffron were dried at room temperature and powdered in a grinder. Lotions containing 2, 4 and 8% of saffron were prepared by the same formulation and procedure of homosalate lotion reference except that homosalate was prepared by saffron**. **

*Sample preparation and determination of SPF*

All samples (1 g) were weighed, transferred to a 100 mL volumetric flask, diluted to volume with ethanol, mixed for 5 min, and then filtered through Whatman filters. A 5.0 mL sample was transferred to a 25 mL volumetric flask and diluted to volume with ethanol. The absorption values were obtained in the range of 290 to 320 nm (every 5 nm) and 3 determinations were made at each point. Then, Mansur equation was used to determine the SPF values of the formulations. Mansur *et al. *(1986) developed a very simple mathematical equation, which in conjunction with substitutes the *in vitro *method proposed by Sayre *et al.*, (1979), UV spectrophotometry. The introduced equation is as follows: 

$\mathrm{SPF}=\mathrm{CF}\times \sum_{320}^{290}\mathrm{EE}.I_{\left(\lambda \right)}.{\mathrm{abc}}_{\left(\lambda \right)}$

In this equation, CF = 10 (Correction Factor), EE_(λ)_= Erythemogenic Effect of radiation at wavelength λ, I_(λ)_ = Intensity of solar light at wavelength λ, and abs_(λ)_ = absorbance of wavelength λ by a solution of the preparation. The homosalate lotion reference containing 8% homosalate presented an SPF value of 4-5, determined by UV spectrophotometry (32, 33). The values for the term “EE × I” are constants, which were determined by Sayre *et al. *(1979), and are shown in Table 1 (34). It was the same procedure to determine homosalate reference (4). 

**Table 1:** The values of “ EE _(λ)_ . I _(λ)_” for conclusion of the SPF values in transmittance method

**λ (nm) **	**EE**_(λ )_ **. I**_(λ)_
290	0.0150
295	0.0817
300	0.2874
305	0.3278
310	0.1864
315	0.0839
320	0.0180

*Moisture content measurement of the skin*

The moisture content of the skin was measured by corneometer (Courage & Khazaka, Cologne, Germany). In practice, the technique is used to measure the difference in the hydration state of stratum corneum before and after application of a cosmetic or other skin treatments. The test was carried out on six volunteers with normal skin, aged between 20 and 35 at room temperature. Before the measurements, they were given time to adapt to the room conditions without covering the measuring sites with clothes. On the day of examination, the skin was not washed and nothing was applied to the skin surface. The volunteers were instructed not to apply any preparation to the site to be examined one week before the investigation. The corneometer CM 820 was used to determine the moisture content of stratum corneum by measuring the electrical capacitance. In all subjects, the tested sites of the skin were free of eczematous involvement. All measured values were expressed as the median of three recordings. The measurements were carried out on exactly the same sites. There is a location-dependent change in the SC hydration state; therefore, the same site of the skin was used for measurement in all cases. The testing site of the skin was the middle of the forearm. At first, the moisture content of the skin was measured without any application of the products, after which the measurement was carried out in 0.5, 3, 5 and 7 h after application of the 8% saffron lotions and the reference lotion without saffron (27, 28, 31).

## Results

In this study, three concentrations of saffron lotions were evaluated by UV spectrophotometry using Mansur mathematical equation (32). 

**Figure 1:**
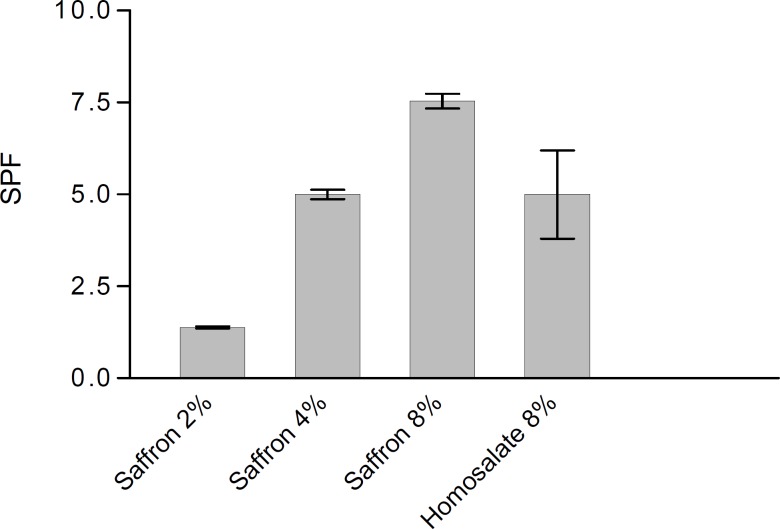
The SPF values of saffron lotions (2, 4 and 8%) and homosalate lotion reference determined by *in vitro *method (n=3).

The SPF values of the 8% homosalate lotion reference and the lotions with 2.0, 4.0 and 8.0% saffron were measured. Figure 1 shows the SPFs of saffron lotions ethanol and homosalate reference. According to this method, the SPF of homosalate lotion reference was calculated as 5.00 ± 1.20, which had no significant difference between our *in vitro *study value and claimed SPF for this standard lotion (4.47 ± 1.279, P > 0.05) (29, 30).

This study showed that there were no significant differences in the SPF values of 4% saffron lotion and 8% homosalate lotion reference. However, the SPF of 8% saffron lotion was significantly higher than 8% homosalate lotion reference (P < 0.05).These results show that in equal concentration, saffron can act as a better antisolar agent compared to homosalate. 

**Figure 2:**
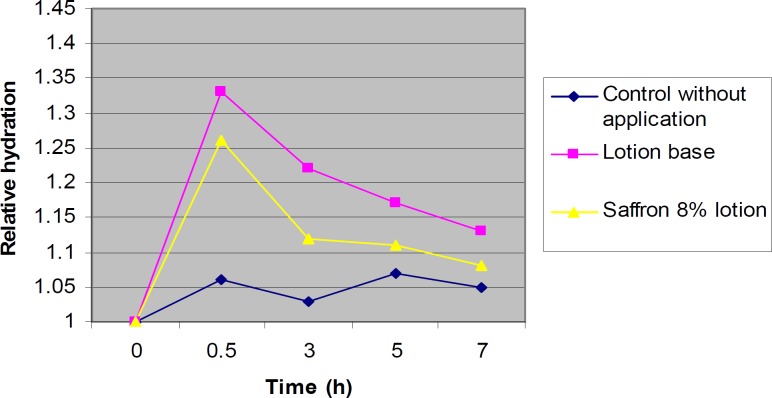
Relative hydration values after application of the lotion without saffron, the 8% saffron lotion in relation to the control (with no treatment) (n = 6).

The lotions containing 8% saffron and the lotion base without saffron were used in our experiments and the water contents were measured using corneumeter CM 825. The corneometer was calibrated to the base line value for each subject before application of the formulations on the skin. During the first 30 minutes after application, the water contents were usually higher than normal. Measurement of the water content of skin at this time may result in erroneous data (27, 31, 35). Therefore, the first measurement was scheduled at 30 min after application. The water content of skin was measured 0.5, 3, 5 and 7 h post-application of 8% saffron lotions, compared to the baseline which was the value before application of the product. There were no significant differences (P > 0.05) between the skin moisture contents after application of saffron lotions and the control lotion (without saffron) during 7 h (Figure 2). Figure 2 shows the normalized hydration values (RCU, Relative Corneometer Units) for the readings of the test sites measured with corneometer for both formulations at 0.5, 3, 5, and 7 h post application in relation to the baseline (the control without treatment). 

## Discussion

The decrease in intensity of the UV radiation reaching the skin by sunscreens may reduce the risk of sun-induced skin cancer (36). The efficacy of a sunscreen is usually expressed by the sun protection factor which is defined as the UV energy required to produce a minimal erythema response after 16-24 h of exposure on protected skin, divided by the UV energy required produce the same degree of erythema on unprotected skin after the same time (2, 3, 31). 

In this study, the homosalate lotion reference and the lotions containing 2, 4 and 8 % saffron were prepared. The SPF values of the formulations were determined by an *in vitro *method according to Mansur *et al *(32). This study indicated that there was no significant difference in the SPF values of 4% saffron lotion and 8% homosalate lotion reference. However, the SPF of 8% saffron lotion was significantly higher than that of homosalate lotion reference. These results showed that in equal concentrations saffron can act better than homosalate as an antisolar agent.

Most of the published studies on determination of the SPF adopt an *in vivo *method based on experiments on human skin, which is very time-consuming and expensive. Therefore, developing an *in vitro *method correlated well with *in vivo *methods is of interest to researchers as an attempt to find a substitute for *in vivo *methods (2, 3, 37). The *in vitro *SPF tests are useful for screening test during product development as a supplement to the *in vivo *SPF measurement. However, it is necessary to standardize these methods to accurately determine the SPF values (37, 38). The *in vitro *methods are in general of two types: methods which involve measurement of the absorption or transmission of UV radiation through the sunscreen films on quartz plates or biomembranes, and methods in which the absorption of dilute solutions of the sunscreens are determined and analysed (4, 32, 33, 39, 40). In the dilution method, different concentrations of the test products in methanol or ethanol are prepared. Each sample’s transmittance is then measured to evaluate the SPF value. 

In this study for determination of the SPF we used the method which was developed by Mansur *et al. *in 1986 (4, 32). He developed a very simple mathematical equation in conjunction with UV spectrophotometry which substitutes the *in vitro *method proposed by Sayre *et al. *(1979) (34). Regarding sun-care experiments, it is also a safety issue, since only positive *in vitro *responses will direct the future of the *in vivo *tests (41). The proposed UV spectrophotometric method is simple, rapid, uses low cost reagents and can be used for *in vitro *determination of SPF values in many cosmetic formulations. It can be performed both during production process, on final product (4).

In recent years, natural compounds or bioactive products have gained considerable attention as UV protective agents due to the presumable safe utilization, ecological issues, and minimum side effects besides their antioxidant activity (42, 43). Plant extracts, due to containing a wide range of phenolic acids, flavonoids, and high molecular weight polyphenols, usually cover the full range of UV wavelengths (42, 44, 45). 

Saffron has been shown to be a source of bioactive compounds with cytotoxic, antitumoral, chemopreventive, antimutagenic and immuno-stimulating properties (46). In a recent review, Abdullaev and Espinosa-Aguirre discussed the experimental *in vitro *and *in vivo *investigations focused on the anticancer activity of saffron and its principal ingredients (18). It was suggested that saffron, which contains a high carotenoid concentration, may be a source for antitumor agents (17, 18).

From the results obtained in our study, the high SPF value of saffron’s lotions may be related to the presence of many aromatic and flavonoid compounds such as kaempherol, quercetin in *Crocus sativus*. In addition, this photoprotective effect may be due to the phenolic components such as tannic, gallic, caffeic, cinnamic, chlorogenic, ferulic and vanillic acids in saffron (44)**. **Caffeic acid, and with a greater degree, ferulic acid proved effective in protecting human skin from UVB-induced erythema. Ferulic acid, shown to be a strong UV absorber, is used as a photoprotective agent in a number of skin lotions and sunscreens. These components exist in saffron extracts (42, 47). One approach to protecting human skin against the harmful effects of UV irradiation is to use antioxidants as photoprotectives. These flavonoid and phenolic components in saffron, also having antitumor and antioxidant activities (17, 18, 46), proposed saffron as a natural sunscreen, which is also indicated from the results of this study.

Phenolics may be beneficial in preventing UV-induced oxygen free radical generation and lipid peroxidation, i.e. events involved in pathological states such as photoaging and skin cancer. Phenolics, particularly polyphenols exhibit a wide variety of beneficial biological activities as well as anti-carcinogenic actions. They are considered as powerful antioxidants (42). 

Quercetin in saffron is a promising flavonoid that possesses the highest antioxidant activity among flavonoids. Topical formulations containing quercetin successfully inhibit UVB-induced skin damage in mice (48, 50). This component can also contribute to the UV protection effects of saffron. 

Another flavonoid, silymarin, isolated from the seeds of *Silybum marianum*, also shows protection against sunburn, DNA damage, nonmelanoma skin cancer, and immunosuppression (49, 50). Many flavonoids such as quercetin, luteolin and catechins are better antioxidants than the nutrients vitamin C, vitamin E and β-carotene (51). They are frequently used in skin lotions and sunscreens.

Green tea polyphenols, *Rosa damascene *flower extracts, *Aloe barbadencis *extract, aromatic compounds isolated from lichens and flowering tops of *Dracocephalum moldavica *and *Viola tricolor *are examples of natural substances evaluated for their sunscreen properties (9-14). Topical application of *Culcitium reflexum *extract in the form of a gel proved to be a significant *in vivo *protection against the UV-induced skin erythema in healthy human volunteers. The flavonolic fraction of Sedum telephium leaf extract also appears to possess potent protective effects against UV-induced skin erythema in human volunteers (14, 42). 

In this research, the water contents of the skin were measured by corneometer (CM 825) after 0.5, 3, 5 and 7 h post-application of the 8% saffron lotion and lotion without saffron, the control being the sample without application of the product (Figure 2). Various methods have been summarized by Fluhr *et al*. (52) for measuring the hydration state of the SC (Stratum Corneum). Common techniques for evaluating moisturizer efficacy are as follows: visual techniques (photography, videomicroscopy, expert visual grading and subject self-assessment), skin hydration measurement (corneometer), skin barrier function (transepidermal water loss measurement) and skin elasticity studies. Among these tests corneometer has been used more widely (53). 

The results obtained show that there were no significant differences in skin moisture contents between 7 h after application of the 8% saffron lotion or the control lotion without saffron, during 7 h. It was observed that for each formulation, there wa as significant increase in moisture content after 30 min. After 1 h, the moisture contents were decreased in all of the formulations. All of the increases in water content after application of the formulations were significantly more than that of the control. The shapes of the curves in all samples were nearly the same. 

There was no significant difference in skin moisture content between the groups applying the saffron lotion or the control lotion without saffron. This indicates that the increase in water content is due to the lotion base and saffron does not have any moisturizing effect. This effect is probably due to the occlusive film on the skin rather than an improvement in the SC regeneration. Some moisturizers are designed to promote water retention by their hygroscopic nature while others are designed to prevent water loss from the skin surface by providing an occlusive film or by supplying SC-like lipids (54). The lack of any remarkable moisturizing effect in saffron may be due to the low lipid content and the absence of hygroscopic components. In lotion base, the occlusive properties of lanolin and white petrolatum, the lipid characteristics of stearic acid, and the hygroscopic effects of propylene glycol may contribute to the increase in water content of the skin. 

## Conclusions

The results of this study indicated that saffron can be used as a natural UV-absorbing agent. The 4% concentration of saffron produced on SPF value equivalent to 8% homosalate lotion reference sunscreen by an *in vitro *method. There was no significant difference between the skin moisture contents after application of the saffron lotions or the control lotion without saffron**. **
